# Evaluation of Homocysteine and Gamma-Glutamyl Transferase Concentrations As Markers of Chronic Kidney Disease: An Indian Perspective

**DOI:** 10.7759/cureus.22959

**Published:** 2022-03-08

**Authors:** Shyamkrishnan R, Gautom K Saharia, Sandip Panda, Manaswini Mangaraj

**Affiliations:** 1 Biochemistry, All India Institute of Medical Sciences, Bhubaneswar, Bhubaneswar, IND; 2 Nephrology, All India Institute of Medical Sciences, Bhubaneswar, Bhubaneswar, IND

**Keywords:** chronic kidney disease, gamma-glutamyl transferase, homocysteine, glomerular filtration rate, end-stage renal disease

## Abstract

Background

Chronic kidney disease (CKD) involves a gradual loss of kidney function over months to years. Oxidative stress plays a critical role in the pathogenesis of CKD. Homocysteine (Hcy), an amino acid derivative, is a known risk factor for oxidative stress and endothelial damage. Gamma-glutamyl transferase (GGT), an enzyme abundant on the cell surface of liver and kidney cells, is raised during oxidative stress. The objectives of this study were to estimate the concentrations of serum Hcy and GGT among CKD patients and healthy controls and to determine whether there is an association between serum Hcy and GGT levels in CKD.

Methodology

A total of 246 participants were needed to meet the calculated sample size. A total of 123 CKD patients meeting the inclusion and exclusion criteria were recruited as cases from the Nephrology outpatient department of our institute. Equal numbers of age- and sex-matched healthy volunteers were recruited as controls. Biophysical profiling of participants was done. Baseline investigations were recorded. A blood sample was collected from each participant and analyzed for GGT and Hcy along with other routine parameters.

Results

Hcy and GGT concentrations were significantly high in CKD patients compared to healthy controls. There was a significant positive correlation between serum GGT and Hcy levels (r = 0.357).

Conclusions

Elevated levels of GGT and Hcy in CKD patients compared to healthy controls demonstrated the oxidative stress associated with the disease. GGT and Hcy can be used as prognostic markers of the disease.

## Introduction

Chronic kidney disease (CKD) includes a spectrum of various pathophysiologic processes culminating in abnormal kidney function with a progressive decline in glomerular filtration rate (GFR). Diabetes mellitus, hypertension, advanced age, autoimmune diseases, previous episode of acute kidney injury, etc., are major risk factors for CKD. Kidney Disease Improving Global Outcome (KDIGO), in its 2012 guidelines, classified CKD into five stages based on the estimated glomerular filtration rate (eGFR) values and albuminuria status. The term end-stage renal disease, which is stage 5 in the KDIGO classification, represents a stage of CKD wherein the accumulation of toxins and electrolytes normally excreted by the kidneys results in uremic syndrome. This syndrome leads to death unless the toxins are removed by renal replacement therapy, using dialysis, or kidney transplantation [1].

The major causes of death in CKD patients are atherosclerosis and cardiovascular diseases (CVDs), which are associated with oxidative stress due to the production of reactive oxygen species (ROS) and reactive nitrogen species (RNS). The oxidative link of these free radicals in kidney disease might be due to mechanisms such as uremic endothelial nitric oxide synthase uncoupling, increased nicotinamide adenine dinucleotide phosphate-oxidases (NADPH oxidases (NOX)) activity, or antioxidant deficit due to dietary restrictions, loss due to diuretics, or malabsorption [2]. These free radicals are known to deteriorate kidney damage by worsening inflammation, activation of nuclear factor-κB (NF-κB), and induction of apoptosis, necrosis, and fibrosis of renal tissue, with increased incidence of amyloidosis due to structural changes in proteins such as β2-microglobulin [3].

Homocysteine (Hcy), an amino acid derived from methionine metabolism, is known to cause atherosclerosis by damaging the endothelium and promoting clotting [4]. The primary cause of Hcy-related pathogenesis is redox imbalance leading to oxidative stress as the molecule is known to bind free Cu^2+^ and impair the functioning of enzymes like superoxide dismutase (SOD). Hcy by themselves can form thiol-thiyl radicals (•RS) leading to ROS production and are known to activate free radical-producing enzymes such as NADPH oxidase [5]. In kidneys, they cause oxidative injury to vascular endothelial cells, thereby contributing to intrarenal arteriosclerosis, reduction in renal perfusion pressure, and, eventually, reduction in eGFR, further worsening the disease [6]. Hence, Hcy can be a marker of the redox status in CKD patients. The reference interval for serum Hcy in healthy adults is less than 15 µmol/L [7].

Gamma-glutamyl transferase (GGT), an enzyme present in serum and on the outer surface of cells from different organs such as the liver, pancreas, intestine, lungs, and kidneys, functions to transfer amino acids across the plasma membrane [8]. In the kidneys, it is mainly located at the proximal tubule and Henle loop [9]. Serum GGT, which is considered a marker of alcohol consumption and hepatobiliary diseases, plays a significant role in the extracellular catabolism of glutathione (GSH), the main antioxidant in mammalian cells [10]. Studies have shown an association between elevated serum GGT levels and complications in diabetes mellitus, CVD, hypertension, and metabolic syndrome. GGT level has been suggested as a predictor of mortality in the general population and can be an early and sensitive marker of oxidative stress even when within the reference interval of 0-30 U/L [8]. Given the importance of oxidative stress in the progression of CKD, GGT can turn out to be an important marker in the detection and prognosis of the disease.

With this background, our study aimed to estimate the concentrations of serum Hcy and GGT among CKD patients for their utility as markers of the disease and compare them with healthy controls. In addition, the study aimed to find out whether there is an association between serum Hcy and GGT levels in CKD.

## Materials and methods

Study design

This was a hospital-based, cross-sectional study conducted in the Department of Biochemistry and the Department of Nephrology, All India Institute of Medical Sciences (AIIMS), Bhubaneswar. The study followed the guidelines included in the Declaration of Helsinki and Tokyo and was granted ethical clearance by the Institutional Ethical Committee of AIIMS, Bhubaneswar (approval number: T/IM-NF/Biochem/20/83 dated August 21, 2020). The recruitment of participants and analysis of the samples was done between October 2020 and September 2021.

Study population

A sample size of 246 was estimated, with 123 participants in each group, that is, patients with CKD and healthy controls at a power of 80% and 95% confidence interval. Individuals of both genders aged above 18 years were included in the study. Patients with a history of alcohol use disorder defined as per the fifth edition of the Diagnostic and Statistical Manual of Mental Disorders, urinary tract infection, acute illnesses, cardiac abnormalities, liver diseases, malignancies, autoimmune disorders, pregnant females, and those with a history of drug abuse that can affect renal function were excluded from the study.

In total, 123 (group A) individuals with a diagnosis of CKD were enrolled from the Department of Nephrology, AIIMS, Bhubaneswar, after obtaining informed written consent. GFR estimation was done using the CKD-EPI creatinine equation, and staging of CKD was done using the KDIGO 2012 guidelines [11]. Group B included 123 age- and sex-matched healthy hospital staff and patient attendants volunteering to participate in the study who were enrolled after clinical examination and renal function tests, with informed written consent from AIIMS, Bhubaneswar.

Data collection

The venous blood sample was collected from each study participant after obtaining informed consent and analyzed on the same day for routine investigations. Samples were collected from the cases before dialysis (predialysis samples) for patients undergoing dialysis.

Hemoglobin estimation was done in the hematology laboratory, AIIMS, Bhubaneswar, on XT 4000-I (Sysmex, Kobe, Japan) fully automated analyzer using anticoagulated 2 mL venous blood sample collected in lavender-capped (EDTA) vacutainers. AU5800 autoanalyzer (Beckman Coulter, Brea, CA, USA) was used for assessing biochemical parameters. For the assessment of renal function, urea was estimated by the urease method, creatinine by the alkaline picrate method, and uric acid by the uricase method. Sodium, potassium, and chloride were estimated by the indirect ion-selective electrode method. Similarly, to assess the liver function, direct bilirubin was estimated by the diazotization method, total bilirubin by the diazotization method with added caffeine, total protein by the biuret method, and albumin by the bromo cresol green method. Aspartate transaminase (AST) and alanine transaminase (ALT) were estimated by the ultraviolet kinetic method using 2-oxoglutarate and alkaline phosphatase (ALP) with the para-nitrophenyl phosphate (PNP) method. Fasting plasma glucose (FPG) was estimated by the hexokinase method, and hemoglobin A1c (HbA1c) by the turbidimetric immunoinhibition method. Estimation of Hcy (using cystathionine beta-synthase) and serum GGT (modified Szasz procedure) were done with AU 480 autoanalyzer (Beckman Coulter, Brea, CA, USA).

Statistical analysis

Data were analyzed using SPSS version 25.0. The skewness of the data was assessed using the Shapiro-Wilk test. It was found to be non-normally distributed. The data were represented as the median and interquartile range (IQR) or number (percentage), wherever appropriate. The comparison of medians was done using the Mann-Whitney U test. The odds ratio was calculated to assess the risk of raised GGT or Hcy levels between CKD subgroups using the logistic regression analysis. Correlation studies between Hcy and GGT were done using Spearman’s rho correlation study. The receiver operating characteristic (ROC) curve for GGT and Hcy were plotted to determine the diagnostic accuracy of the parameters. P-values of <0.05 were considered significant for all statistical methods.

## Results

Baseline characteristics

The tables present the results of the cross-sectional study, which included a total of 246 participants. Of the 123 CKD patients, 102 belonged to the stage 5 category, 14 belonged to stage 4 CKD, and seven were stage 3, according to the KDIGO classification, as expected in a tertiary care center. The basic demographic data of the study group in Table 1 show that there was no significant difference in age or sex between the two groups. The body mass index and the waist-hip ratio of CKD patients were significantly lower than healthy controls (p < 0.001). Systolic and diastolic blood pressures of CKD patients were significantly higher than healthy controls (p < 0.001).

**Table 1:** Baseline characteristics of the study groups. Results represented as median (IQR) or the number of subjects (percentage). *P-values of <0.05 show a statistically significant difference. P-values were calculated by the Mann-Whitney U test for continuous data and the chi-square test for categorical data. BMI: body mass index; BP: blood pressure; IQR: interquartile range

Parameters	Patients with chronic kidney disease (n = 123)	Healthy controls (n = 123)	P-value
Age (years)	46 (19)	48 (20)	0.881
Males	91 (74%)	91 (74%)	1.000
Females	32 (26%)	32 (26%)	1.000
Participants with diabetes	43 (35%)	Nil	NA
Participants with hypertension	72 (58%)	Nil	NA
BMI (kg/m^2^)	20.6 (4.8)	25.3 (2.7)	<0.001*
Waist/Hip ratio	0.90 (0.04)	0.91 (0.03)	<0.001*
Systolic BP (mmHg)	154 (36)	120 (10)	<0.001*
Diastolic BP (mmHg)	90 (20)	80 (10)	<0.001*

The baseline biochemical parameters of urea, creatinine, and potassium levels were significantly higher in CKD patients compared to healthy controls (p < 0.001 for each), with significantly lower sodium levels (p < 0.001). Total bilirubin, AST, ALT, total protein, and albumin were significantly lower in CKD patients (p < 0.001). Hemoglobin level was found to be significantly low in the CKD group with a p-value of <0.001.

**Table 2:** Comparison of biochemical parameters between patients with chronic kidney disease and healthy controls. Results represented as median (IQR). *P-values of <0.05. P-values were calculated using the Mann-Whitney U test. IQR: interquartile range

Parameters	Patients with chronic kidney disease (n = 123)	Healthy controls (n = 123)	P-value
Urea (mg/dL)	114 (58)	19 (8)	<0.001*
Creatinine (mg/dL)	7.5 (4)	0.7 (0.2)	<0.001*
Uric acid (mg/dL)	7.4 (2.7)	4.4 (1.5)	<0.001*
Sodium (meq/L)	136 (5)	138 (3)	<0.001*
Potassium (meq/L)	4.8 (1.4)	4.1 (0.3)	<0.001*
Chloride (meq/L)	101 (8)	101 (4)	0.501
Total bilirubin (mg/dL)	0.5 (0.3)	0.6 (0.3)	<0.001*
Direct bilirubin (mg/dL)	0.1 (0.01)	0.1 (0.01)	0.556
Aspartate transaminase (IU/L)	20 (11)	22 (9)	<0.001*
Alanine transaminase (IU/L)	15 (12.8)	21 (18)	<0.001*
Alkaline phosphatase (IU/L)	120 (47)	86 (34)	<0.001*
Total protein (g/dL)	7 (1.1)	7.7 (0.8)	<0.001*
Albumin (g/dL)	3.8 (0.8)	4.3 (0.6)	<0.001*

**Figure 1:**
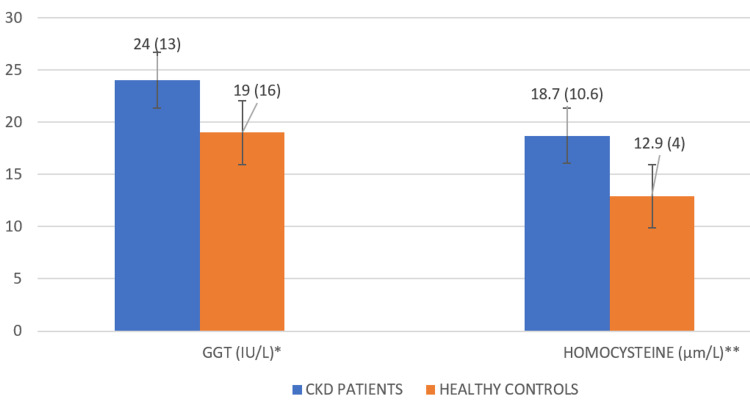
Comparison of serum GGT and Hcy between patients with chronic kidney disease and healthy controls. Results represented as median (IQR). *P-values = 0.007; **P-values < 0.001. GGT: gamma-glutamyl transferase; Hcy: homocysteine; IQR: interquartile range

Both serum GGT and Hcy registered a prominent rise in CKD patients compared to healthy controls (p < 0.05), as shown in Figure 1.

Odds ratio analysis of the parameters in CKD subgroups, that is, those with and without diabetes and those with and without hypertension, noted no significant difference in risk (Table 3).

**Table 3:** Odds ratio analysis for GGT and Hcy levels among different subgroups of CKD. Results represented as odds ratio with confidence interval and p-values. Odds ratio calculated by logistic regression analysis. CKD: chronic kidney disease; T2DM: type 2 diabetes mellitus; Hcy: homocysteine; GGT: gamma-glutamyl transferase

CKD patients with T2DM (n = 43) and CKD patients without T2DM (n = 80)			
Parameter	Odds ratio	95% confidence interval	P-value
Hcy (µmol/L)	0.977	0.941-1.014	0.224
GGT (IU/L)	1.016	0.994-1.040	0.158
CKD patients with hypertension (n = 72) and CKD patients without hypertension (n = 51)			
Parameter	Odds ratio	95% confidence interval	P-value
Hcy (µmol/L)	0.987	0.963-1.013	0.321
GGT (IU/L)	1.006	0.990-1.024	0.455

Receiver operative characteristic curve

**Figure 2:**
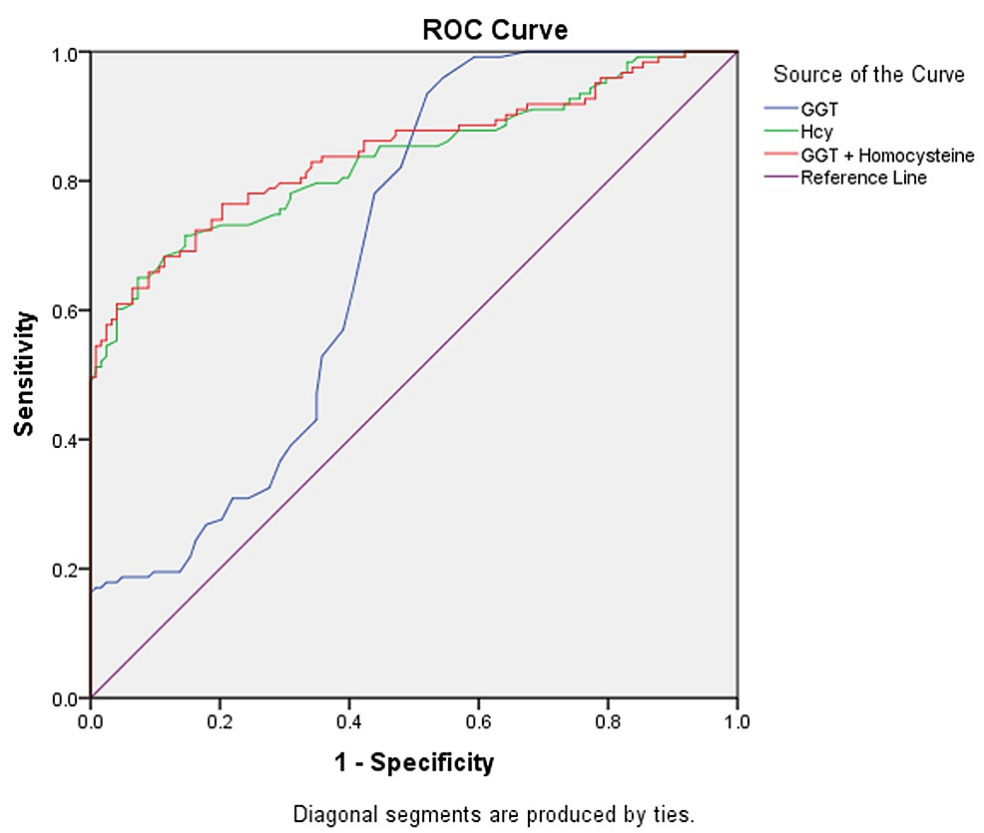
ROC curve for serum Hcy and GGT levels for predicting CKD. AUC for Hcy is 0.833 (p < 0.001), for GGT 0.692 (p < 0.001), and with use of both markers combined is 0.844 (p < 0.001). The cut-off value for Hcy as a marker for detection of late stages of CKD is 14 µm/L (sensitivity of 80% and specificity of 70%), and that for GGT is 20.5 IU/L (sensitivity of 80% and specificity of 60%). ROC: receiver operative characteristic; Hcy: homocysteine; GGT: gamma-glutamyl transferase; CKD: chronic kidney disease; AUC: area under the curve

ROC curve for Hcy and GGT was plotted. The area under the curve (AUC) for Hcy was 0.833 (p < 0.001), GGT was 0.692 (p < 0.001), and with both combined was 0.844 (p < 0.001), showing their usefulness as prognostic markers of late stages of CKD, as shown in Figure 2.

**Figure 3:**
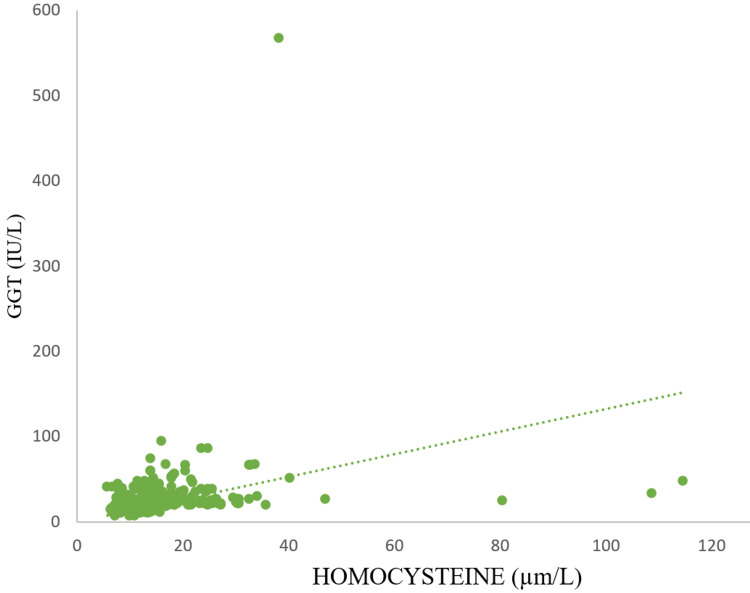
Correlation of GGT levels with Hcy levels. Spearman’s rho correlation coefficient r = 0.357, with p-value of <0.001. Hcy: homocysteine; GGT: gamma-glutamyl transferase

The cut-off value obtained from the study for the detection of late stages of CKD for Hcy was 14 µm/L, with a sensitivity of 80% and specificity of 70%, and that for GGT was 20.5 IU/L, with a sensitivity of 80% and specificity of 60%. A significant positive Spearman’s rho correlation was observed between serum Hcy and GGT (r = 0.357), revealing their association in CKD (Figure 3).

## Discussion

The present study was conducted to evaluate the serum levels of Hcy and GGT in patients with CKD, involving a total of 246 study participants (123 CKD patients and 123 controls).

In participants with CKD, the features characteristic of the disease were high serum urea, creatinine, uric acid, and potassium with low sodium levels. The significantly low levels of total protein and albumin are due to proteinuria, especially albuminuria, associated with CKD [12]. The levels of serum AST and ALT were significantly low in CKD patients. Ray et al. had previously identified low serum levels of AST and ALT in CKD patients. The possible mechanisms may be due to reduced levels of pyridoxal-5-phosphate, the co-enzyme for transaminases, reduction in synthesis, and release of enzymes from hepatic cells with accelerated clearance, along with the hemodilution and water retention associated with the disease [13]. Hemoglobin levels were significantly lower in patients with CKD. The reduction in the synthesis of erythropoietin related to renal failure is the main reason for this abnormality. Other factors such as increased hemolysis, inflammation, chronic blood loss, nutritional deficiency, hyperparathyroidism, and marrow suppression can also contribute to the disease severity [14].

This study documented significantly higher Hcy levels in CKD patients compared to the healthy control group. The kidney is a major site of Hcy metabolism. Hcy formed in the body can be transsulfurated to cysteine or remethylated to methionine. Both transsulfuration and remethylation routes are used by renal epithelial cells. However, Hcy appears to be predominantly degraded by transsulfuration in the epithelial cells lining the proximal tubule. When clearance is impaired, the level of plasma and tissue Hcy increases, resulting in hyperhomocysteinemia. Hyperhomocysteinemia is a risk factor for CKD. Accumulation of Hcy leads to vasoconstriction and impairment of renal microvasculature. The reduction in renal function, in turn, leads to further accumulation of Hcy, resulting in chronic renal failure leading to a vicious cycle [15]. Other contributory mechanisms of renal damage in hyperhomocysteinemia include antagonizing the vasodilatory action of nitric oxide (NO) by forming S-nitroso Hcy and Hcy-induced glomerulosclerosis with subsequent loss of renal function by phenotypic changes in podocytes [16].

Approximately 3% of end-stage renal diseases are due to ischemic renal injury. Ischemia-reperfusion leads to oxidative stress in the kidney resulting in a marked increase in Hcy. Hence, renal injury after ischemia and ischemia-reperfusion can be attributed to Hcy toxicity. Moreover, Hcy elevations lead to CVDs, which is a leading cause of mortality in end-stage renal disease [5]. A study by Lai et al. found that patients who are overweight/obese with higher Hcy levels have an increased risk of CKD [6]. Yeh et al. also found elevated Hcy levels in patients with CKD [17].

The study also showed significantly high levels of GGT in CKD patients compared to healthy subjects. The ROC curve demonstrated that serum GGT levels could also be a predictor of kidney disease. GGT, as already described, is an enzyme involved in the extracellular metabolism of GSH, the main antioxidant in mammalian cells [10]. When GGT acts upon GSH in the extracellular environment, the gamma-glutamyl group is added to an acceptor amino acid, and cysteinyl glycine is released, which is then acted upon by dipeptidases. The final products, cysteine, and glycine are taken up by the cells and used for de novo GSH synthesis. More often, the acceptor amino acid for GGT would be cysteine, resulting in the formation of gamma-glutamylcysteine, which is taken up by the cells directly and used for GSH synthesis by the salvage pathway. Removal of the gamma-glutamyl group from GSH-S conjugates by GGT is the first step in the conversion of these compounds to mercapturic acid, which is involved in detoxification reactions [18].

Cells are known to respond to oxidative stress by upregulation of GSH and all enzymes involved in its synthesis; hence, increased GGT is indicative of intracellular GSH depletion, and serum levels of GGT may be considered a marker of oxidative stress [19]. Given the role of oxidative stress in the pathogenesis of CKD, the significantly high GGT levels in CKD patients in the study are self-explanatory.

Previously, Chen et al. found that elevated GGT increases the rate of CKD [20]. Shen et al., in their study on the Chinese population, found that there is a positive relationship between increasing serum GGT concentrations and the incidence of CKD, which suggests that elevated GGT could be a potential indicator for risk of CKD [21]. Sun et al. studied the relation between serum GGT and albuminuria, another indicator of kidney damage, and found that serum GGT level is associated with urinary albumin excretion in middle-aged and elderly Chinese [22]. Ryu et al. evaluated GGT levels of 10,337 non-hypertensive and non-diabetic males in Korea, demonstrating an increased risk for CKD with an increasing quartile of serum GGT [23].

Odds ratio analysis of serum Hcy and GGT levels between different subgroups of CKD, that is, CKD patients with and without diabetes and CKD patients with and without hypertension, showed no significant difference in risk. This points out that the rise of these parameters in CKD patients is related to the disease progression, irrespective of their diabetic or hypertensive status.

Spearman’s rho correlation analysis showed a significant positive correlation between serum Hcy and GGT levels in our study. A mechanism relating to the serum Hcy levels and molecules related to redox balance has been postulated. The methionine cycle and transsulfuration pathway of Hcy metabolism regulate cellular redox state by modulating GSH levels. Hcy inhibits the enzyme cystathionine β synthase, which is involved in the synthetic pathway of cysteine from methionine. At high concentrations, Hcy also inhibits the enzyme cystathionine lyase, another enzyme of the cysteine synthetic pathway. As cysteine is necessary for GSH synthesis, hyperhomocysteinemia leads to decreased levels of GSH [5]. Sen et al. identified that accumulation of Hcy was associated with enhanced oxidative stress characterized by increased superoxide (O^2-^) and oxidized GSH [24]. This depletion of GSH can have an impact on the levels of GGT in patients with CKD leading to their subsequent increase.

Studies are yet to be conducted in the Indian population to evaluate the levels of serum Hcy and GGT in CKD patients, and to our knowledge, this is the first study to assess both Hcy and GGT levels in these groups of patients. It has to be noted that, despite other liver enzymes being significantly low due to hemodilution, the high levels of GGT and Hcy could be highlighted. This opens the possibility of using these molecules as markers, as established in the ROC curves, for assessing the severity and prognosis of the disease. Future studies with antioxidant supplementation and assessing the levels of Hcy and GGT after treatment would give further insight into the condition. Moreover, Hcy and GGT estimation could possibly predict cardiovascular and other adverse outcomes associated with CKD, which has to be established through further prospective studies.

As the study was conducted in a tertiary care center, the participants belonged to stages 3, 4, and 5 of the KDIGO classification. Hence, the utility of these markers in the early stages could not be evaluated, which is a limitation of our study. Due to the limited number of participants with stages 3 and 4 of CKD in the tertiary care institution, the stage-wise comparison could also not be attempted.

## Conclusions

This is the first study of its kind from India comparing serum Hcy and GGT among CKD patients and healthy controls. We tried to find the association of serum Hcy and GGT levels in CKD. Elevated serum Hcy and GGT were seen in the serum of CKD patients compared to healthy controls, highlighting the role of oxidative stress in the morbidity and mortality associated with CKD. Hence, steps may be taken to reduce the oxidant status with prophylactic antioxidant nutrients for clinical improvement of the cases and to slow down the rapid progression of disease with deterioration of kidney function. Prospective studies can be conducted to observe the clinical improvement with antioxidant measures.
